# The Ketogenic and Atkins Diets Effect on Intractable Epilepsy: A Comparison

**Published:** 2014

**Authors:** Ahad GHAZAVI, Seyed Hassan TONEKABONI, Parvaneh KARIMZADEH, Ahmad Ali NIKIBAKHSH, Ali KHAJEH, Afshin FAYYAZI

**Affiliations:** 1 Neurophysiology Research Center, Department of Pediatric Neurology, Urmia University of Medical Sciences, Urmia, Iran; 2 Pediatric Neurology Research Center, Shahid Beheshti University of Medical Sciences, Tehran, Iran; 3 Pediatric Neurology Center of Excellence, Department of Pediatric Neurology, Mofid Children Hospital, Faculty of Medicine, Shahid Beheshti University of Medical Sciences, Tehran, Iran; 4 Department of Pediatric Nephrology, Urmia University of Medical Sciences, Urmia, Iran; 5 Department of Pediatric Neurology, Zahedan University of Medical Sciences, Zahedan, Iran; 6 Department of Pediatric Neurology, Hamedan University of Medical Sciences, Hamedan, Iran

**Keywords:** Classic ketogenic diet, Atkins diet, Intractable epilepsy

## Abstract

**Objective:**

Intractable epilepsy is a major difficulty in child neurology, because the numbers of drugs that are available for treatment are limited and new treatments such as diets must be tried. Now there are some diets available for treating patients with intractable epilepsy. The oldest diet is the classic ketogenic diet and one of the newest diets is the modified Atkins diet. Patients have a harder time accepting the classic ketogenic diet than the Atkins diet, which is easier to accept because the food tastes better. This study compares the efficacy of the ketogenic diet and the Atkins diet for intractable epilepsy in children.

**Materials & Methods:**

This study is a clinical trial survey with sample size of 40 children with refractory epilepsy who were patients at Mofid hospital in Tehran, Iran. Initially, from Jan 2005–Oct 2007, 20 children were treated with the Atkins diet, and then from Oct 2007–March 2010, the other group was treated with the classic ketogenic diet and the results were compared.

**Results:**

In this study, response to treatment was greater than a 50% reduction in seizures and at the end of first, second, and third months for the ketogenic diet were 55%, 30%, and 70% and for the Atkins diet were 50%, 65%, and 70%, respectively.

**Conclusion:**

The results of this study show that there is no significant difference between the classic Ketogenic diet and the Atkins diet at the end of first, second, and third months and both had similar responses to the treatments.

## Introduction

Epilepsy has a 120/100000 incidence ratio in the first year of life and an incidence ratio of 40–50/100000 in 1- to 10-year-old children (1). This disease is the most common reason for referral to neurology clinics. A total of 15–20% of the epileptic cases is resistant to drug therapy (2).

Refractory epilepsy is one of the true nuisances in child neurology that causes increasing annual costs for treatment (3).

There is no agreed upon definition of refractory epilepsy among the authorities. Many physicians believe that refractory epilepsy applies to the type of epilepsy that is not controlled with three to four appropriate medications with an acceptable dosage and proper duration (3). Persistent attacks may lead to irrevocable complications the brain. Currently, the treatment methods for this problem such as surgery, vagus nerve stimulation, new medications, and the Ketogenic diet are costly except for the Ketogenic diet. The Ketogenic diet has been applied in neurology for at least90 years. In 1910, Gopa and Marie published a report concerning the impact of fasting as a treatment for epilepsy, but since fasting was impractical, it was largely forgotten. In 1972, Livingstone of the John Hopkins Center cured approximately 1000 epileptic patients with the Ketogenic diet and later, Freeman (from Johns Hopkins as well) continued Livingstone’s work. Today the Ketogenic diet is a standard treatment for refractory epilepsy (4–7). In the Ketogenic diet, the daily calorie intake is reduced by75% and 1gm/kg protein and 5–10 gm carbohydrate is recommended with the remainder of daily calorie intake provided by fat (4).

Researchers have suggested some alteration to the diet to make it more practical and acceptable for children.

The Atkins diet is the diet of last resort. This diet was introduced by Robert Atkins in 1970 for the prevention of obesity. In this diet, just as in the Ketogenic diet, carbohydrates are restricted and fat is increased to result in weight loss. If the amount of carbohydrates restricted is sufficient to cause ketosis in patients, then it may lead to a decrease of epileptic attacks in refractory epileptic children (4).

In the Atkins diet, severe restrictions on daily calorie consumption and protein are unnecessary. We intend to evaluate the effects of the classic Ketogenic diet and Atkins diet on children with refractory epilepsy and compare the decreases in epileptic attacks.

## Materials & Methods

**Definitions**

**Refractory Epilepsy: **Epilepsies that are not cured by at least three first line anti-epileptic medications (barbiturates, benzodiazepines, phenytoin, carbamazepine, sodium valproate, and/or ethusuximide) with accurate indications, appropriate dosages, and sufficient durations (3).

**Atkins Diet: **A diet that suggests 60% of the necessary daily calorie intake is provided by fat, 30% by protein, and limited to 10 gm of carbohydrates (4).

**Ketogenic Diet: **A diet that suggests the daily calorie intake is reduced by75%. The daily calorie intake is provided by 1 gm/kg of protein and 5–10 gm of carbohydrates and the remainder of the calorie intake is provided by fat (4).

**Response to treatment: **Reductions of greater than 50% in the number of daily epileptic attacks after starting the diets.

**Enrolled Criteria**

All 1- to 16-year-old patients who suffered from refractory epilepsy within the criteria listed below:

1. No febrile convulsions;

2. Without brain neoplasm and progressive neurologic diseases; and,

3. Children of mothers with at least primary school education.

In this study, from January 2005–October 2007, 20 patients aged1 to 16 years of age with refractory epilepsy who were referred to the neurology clinic of Mofid hospital in Tehran, Iran and met the above criteria for the study were treated with the Atkins diet. Alternatively, from October 2007–March 2010, another 20 patients aged 1 to 16 years of age who were similar to the first group of patients for age and frequency of attacks were put on the Ketogenic diet. First, the demographic data, parent education and job, the type and number of epileptic attacks, positive clinical manifestations, EEG and neuroimaging, anti-epileptic drugs, and the duration of previous treatments were entered on the evaluation forms. The results of the treatment were recorded after the first, second, and third months.

**Statistical Analysis**

After the data was gathered, a statistical analysis was performed using paired t-test, the Fisher’s exact test, Friedman’s non-parametric, Wilcoxon’s non-parametric test, and repeated measure analysis of variance (ANOVA). SPSS 17 for Windows (SPSS Inc., Chicago, IL) was used for statistical analysis. This study was approved by the ethics committee of Shahid Beheshti University of Medical Sciences. Informed consent was given by the parents of patients in this study.

## Results

Tables 1–5 show the demographic data and the frequency of the variables in both groups. The mean of daily seizures before the Atkin’s diet was 14.15±12.60 (range, 2–50) and for the Ketogenic diet was 15.30±14.67 (range, 2-50). There were no significant differences evident between the mean number of daily attacks before treatment (p=0.862) and shows that they were similar in the number of attacks when the data was compared.

In the Atkin’s diet group after 1-month, 2-months, and 3-months of the diet, a mean value of 7.6±11.47 (range, 0–50), 6.75±11.11 (range, 0–50), and 6.60±11.25 (range, 0–50) daily seizures were reported, respectively. These values were 6.50±8.29 (range, 0–30), 3.20±4.94 (range, 0–20), and 7.5±11.75 (range, 0–35), respectively, for the Ketogenic diet group. We used the Kolmogorov- Smirnov test in which none of the variables showed normal distribution (all four p-values<0.01)to compare the normal distribution of the seizures in each group.

The Wilcoxon non-parametric test was used to compare the mean number of daily attacks before starting the Atkins and the Ketogenic diet with the number of seizures after 1-, 2-, and 3-months of treatment in each group to consider the non-normal distribution for the number of seizures at the different stages. In the Atkins group, after 1-month (p<0.001), after 2-months (p=0.001), and after 3-months (p=0.001) showed significant differences when compared to data from before the diet was started. In the Ketogenic group, after 1-month (p<0.001), after 2-months (p<0.001), and after 3-months (p=0.002) showed significant differences when compared to the data from before the diet was started. These results reveal that both diets are effective in lowering the number of attacks.

The response to treatment, which was a reduction of greater than 50% in the number of daily attacks when compared with data from before treatment; after 1-month of diet treatment: in the Atkins group, 50% of patients and in the Ketogenic group, 55% of the patients showed improvements from the diet treatments.

After 2-months of diet treatment, the success rate was 65% for the Atkins diet group and 30% for the Ketogenic diet group; and after 3-months, 70% of the Atkins diet group, and 70% of the Ketogenic diet group had appropriate responses. Using the Fishers exact test to compare the two groups, a significant difference was not detected between the groups after 1-(p=1), 2-(p=0.127), and 3-months (p=1) and both diets were similarly successful in response to treatment.

After 3-months of diet treatment, 30% of the patients in both groups, which showed reduction of greater than 50% in the frequency of seizures when compared with the data from before the ; had no response to the diets.

A total of 35% of the Atkins diet group and10% of the Ketogenic diet group had a 51–75% decrease in the frequency of seizures.

A 76–99% decrease was detected in 15% of the Atkins diet patients and 30% of the Ketogenic diet patients after 3-months of the therapy. In addition, 20% (4cases) of the Atkins group and 30% (6 cases) of the Ketogenic group indicated no seizures. The Chi-square test indicated that there was no significant difference between the two groups.

There was also no correlation between the age of patients at the start of diet, gender, parent education and job, type of seizures, type of epileptic syndrome, brain imaging findings, EEG findings, and decreases in the frequency of seizures after 3-months therapy in either of the diets.

The follow up duration for these patients for each group was 3-months due to limitations but many patients especially those patients who had a good response and continued the diet as well as all patients who did not respond well from anti-epileptic drugs and the adherence to the diets was good had positive parental response to the diet.

## Discussion

Previous studies have indicated the effectiveness of the Ketogenic diet and to a lesser degree on the Atkins diet, the impact, and assessment separately on refractory epilepsy. Tonekaboni et al. in Iran (8) indicated that after 3-months on the Atkins diet, 66.7% of patients had greater than 50% and 25% had greater than 99% decreases in the frequency of seizures. Furthermore, 20.8% of the patients were seizure-free.

Eric H. Kossoff indicated that the effectiveness of the Atkins diet (9) was45% of patients had 50–90% and 28% of the patients had greater than 90% decreases in the number of seizures.

Susanne Weber et al. in Denmark indicated that the Atkins diet was used for treatment of 15 children with refractory epilepsy. After 3-months, six patients (40%) had greater than a 50% reduction in the number of seizures. After 12-months, only three patients (20%) continued the diet with no significant change in the frequency of seizures.

Mirjavadi et al. showed that the effectiveness of the Ketogenic diet in 66 children in Iran (10) with11 patients not continuing the diet. After 3-months, 71% of the patients had greater than 50% fewer epileptic attacks.

This study is reliable, because of the relevant sample size. Karimzadeh et al. study on 87 children with refractory seizure showed ketogenic diet caused50% reduction in seizure frequency in 87% of patients and 39% of whom showed complete seizure control in the third month (11).

Barzegar et al. (2009)at the Tabriz Children’s hospital, the impact of the Ketogenic diet was evaluated on 28 1.5–14-year-old (mean, 6.8 years) children with refractory epilepsy who had no response to two antiepileptic drug medications (12). These patients had a mean number of 14 seizures a week and were followed up for 6-months. After 1-month of the diet, 42.9% of the children were seizure-free and 28.6% had a greater than 50% decrease in seizure frequency. After 3-months on the diet, 39% were seizure free and 14% had at least a 50% decrease in the frequency of seizures. These figures were 28.63% and 17.9%, respectively after 6-months on the diet. A39% decrease in the number of attacks demonstrates the positive effect of the diet as a treatment for refractory epilepsy.

In our study, at the end of 3-months 70% of the patients in each group with a 50% reduction in the number of seizures showed an appropriate response to the diet.

The results of all mentioned studies that were done with the Ketogenic and Atkins diets separately and

were similar to our study in that they showed a good response to these diets for the reduction in the frequency of seizures.

In a systematic assessment of 11 published articles performed by Naomi Aranson and Frank Lefevre (13) on the impact of the Ketogenic diet as shown below with the results indicated:

A total of 16% of patients were seizure-free, 32% of patients had greater than a 90% decrease, and 56% had greater than a 50% decrease in seizure frequency.

In another systematic review performed by Daniel L. Keene on the Ketogenic diet (14), 14 studies were evaluated and the results showed that 15.6% were seizure-free and 33% of the patients had greater than a 50% decrease in the frequency of seizures.

The results of these two systematic reviews also support our study regarding the anti-epileptic effects of the diets as treatment.

**Table 1:** The Mean Age and Gender Distribution in the Atkins and Ketogenic Diet Groups

**Variables**		**Atkins**	**Ketogenic**	**P-Value**
**Mean age**		78.45±23.85	40.26±17.69	0.286
**Sex**	**Male**	70%(14)	65%(13)	>0.05
	**Female**	30%(6)	35%(7)	

**Table 2:** Fathers’ Job from the Study Groups

Variables		Tradesman	Laborer	Employee	P-Value
Fathers’ Job	Atkins	50%(10)	5%(1)	45%(9)	0.312
	Ketogenic	35(7)%	20(4)%	45(9)%	

**Table 3:** Mothers’ Job in the Study Groups

Variables		With Job	Without Job	P-Value
Mothers’ Job	Atkins	5%(1)	95%(19)	1.000
	Ketogenic	5%(1)	95%(19)	

**Table 4:** Parents’ Education in the Study Groups

Variables		Elementary to Diploma	Up to Licentiate	Beyond Licentiate	P-Value
Fathers’ Education	Atkins	70%(14)	25%(5)	5%(1)	1.000
	Ketogenic	70%(14)	25%(5)	5%(1)	
Mothers’ Education	Atkins	70%(14)	3%(6)	0%(0)	0.344
	Ketogenic	80%(16)	15%(3)	5%(1)	

**Table 5:** Disease Characteristics in Each Study Group

Variables		Atkins	Ketogenic
Syndrome Classification	Idiopathic	20%	5%
	Cryptogenic	25%	45%
	Symptomatic	55%	50%
Seizure Type	Generalized tonic clonic	10%	10%
	Generalized tonic	5%	35%
	Myoclonic	10%	30%
	Complex partial	5%	0%
	Mixed	70%	25%
EEG Abnormality	Spike wave	35%	70
	Sharp wave	90%	45%
	H.V.S.W	55%	15%
	Hypsarrhythmia	10%	5%
Imaging	Normal	45%	50%
	PVL	5%	20%
	Focal Lesion	20%	0%
	Atrophy	30%	10%
	Porencephalic cyst	0%	10%
	Calcification	0%	5%
	Subdural effusion	0%	5%

In our study, a comparison of the two groups using the Fisher exact test did not indicate a significant difference regarding the effectiveness of the diets. Moreover, 30% of the patients in each group did not respond to treatment at all, 35% of the Atkins group and 10% of the Ketogenic group had approximately 51–75% seizure frequency reduction and in 15% of the patients on the Atkins diet and 30% of the patients on the Ketogenic diet showed an approximate76–99% seizure frequency reduction was observed. A total of 20% of the Atkins diet group and 30% of the Ketogenic diet group were seizure-free after 3-months. The Chi-square test showed no significant differences observed between the two groups.

Natach Porta et al. at the Lille University-affiliated Hospital, France (15) showed that these two diets when compared to a smaller sample size of 27 patients, of which 17 were on the Ketogenic diet and 10 were on the Atkins diet for 6-months; the response to treatment was 65% for the Ketogenic diet and 20% for the Atkins diet after 3-months.This indicates a significant difference between the two groups and shows that the Ketogenic diet was more successful. However, after 6-months 41% of the Ketogenic diet group and 20% of the Atkins diet group exhibited responses to their respective diets that demonstrated no significant differences on the antiepileptic effects.

Similar to our study, our results were similar after 6-months and showed that the same effect from the two diets, but the results were different after 3-months.

We think two reasons that explain these differences are as follows: 1.the smaller number of patients in Porta et al (15), and 2.different causes of refractory epilepsy in these two studies.

**In conclusion, **based upon the aforementioned studies, the effectiveness of the Atkins and Ketogenic diets on refractory epilepsy has been defined and we have concluded equal effects from both diets. Since, the Atkins diet is easier to implement and maintain when compared to the Ketogenic diet, i.e., there is no need for hospitalization, no restriction of protein, calories, fluids as well as better tasting food, it may be possible to replace the Ketogenic diet with the Atkins diet for the treatment of refractory epilepsy.

## Author’s contribution

Dr Ghazavi: Writing the article, data collection, statistical analysis, obtained funding, Analysis and interpretation, Final approval of the article

Dr Tonekaboni and Dr Karimzadeh: Conception and design, data collection, analysis and interpretation, final approval of the article, helping in manuscript writing and editing

Dr Fayyazi and Dr Khajeh: Data collection, analysis and interpretation

Dr Nikibakhsh: Writing the article, revision of the article, final approval of the article.
